# A nanoprecursor method for successfully synthesizing clinoptilolite with high-crystallinity and resultant effects on CO_2_/CH_4_ selective adsorption†

**DOI:** 10.1039/d1ra03314j

**Published:** 2021-09-15

**Authors:** Chengwei Zhai, Jihong Sun, Bingying Jia, Anadil Gul, Shiyang Bai

**Affiliations:** Beijing Key Laboratory for Green Catalysis and Separation, Department of Environmental and Chemical Engineering, Beijing University of Technology Beijing 100124 China jhsun@bjut.edu.cn

## Abstract

Nanoprecursors used as a structural promoter (SP) were prepared by a hydrothermal method and named sol-SP. After centrifugation, the supernatant and precipitate were denoted as solution-SP and solid-SP, respectively. The effect of the additive amount on the structures and properties of the synthesized clinoptilolite was investigated using various characterization techniques. The activation energies of crystallization kinetics during induction and growth periods were calculated. The results showed that the induction period is the control step during the synthesis of clinoptilolite, while additive sol-SP or solid-SP was beneficial to shorten the induction period and therefore enhance the formation of the crystal nucleus. When their pre-crystallization time was too long or the additive amount was too much, the impure phase (phillipsite) in the synthesized clinoptilolite was easily generated. Although the addition of solution-SP had no obvious effect on the induction period, it promoted the growth of crystals after nucleation. Finally, the adsorption performances for CO_2_ and CH_4_ were preliminarily assessed using synthetic clinoptilolite as the adsorbent, showing the promising application for the separation of CO_2_/CH_4_.

## Abstract 2

Nanoprecursors used as a structural promoter (SP) were prepared by a hydrothermal method and named sol-SP.

## Graphical abstract

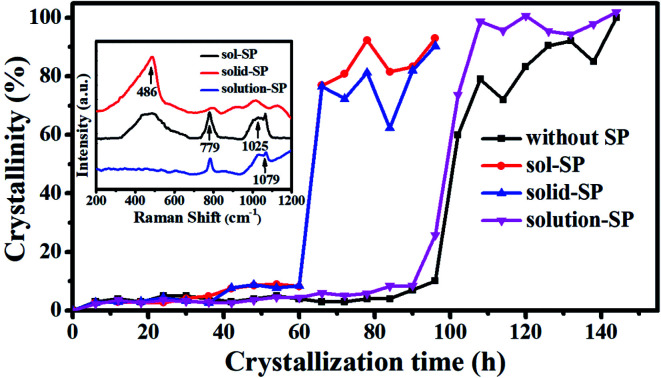

## 1 Introduction

Clinoptilolite (CP) with heulandite (HEU) structure feature is one of the widely distributed zeolite minerals in nature. Because of its large specific surface area, good ion exchange capability, uniform pore diameter, good adsorption property, low cost, and other characteristics, it is commonly used for gas adsorption and separation.^1^ Particularly, the selective performances and adsorption capacities of mixture gas largely depend on the type, number, and the position of the equilibrium cations in its HEU skeletons.^2,3^ Frankiewicz *et al.*^4^ reported that various cations in the presence of HEU skeletons and their distributions would affect the adsorption/separation of CH_4_/N_2_ and their kinetic performances. Earlier, Chao^5^ found that Mg-CPs could definitely improve the separation performance of CH_4_/N_2_ or CH_4_/CO mixture gases. In 2001, Aguilar-Armenta *et al.*^6^ investigated the adsorption rates of pure CO_2_, O_2_, N_2_, and CH_4_ using various cation-exchanged (Ca^2+^, K^+^, and Na^+^) CPs as adsorbents, and further demonstrated that cation-exchanged CPs could be used for the separation of N_2_/O_2_, N_2_/CH_4_, and CO_2_/CH_4_ mixtures. Thereafter, Kouvelos *et al.*^7^ conducted detailed exploration on monovalent ion-exchanged CPs, such as Na^+^ and Li^+^, and their high adsorption capacity and denitrification selectivity further proved them to be promising adsorbents in dynamic pressure swing adsorption separation of CH_4_/N_2_.

However, natural CP usually contains a large number of impurities and miscellaneous phases, easily leading to micropore blockage, and therefore greatly limiting its applications. In this regard, how to synthesize high purity CP is currently one of the main topics.^8^ In 1963, Ames^9^ reported the synthesis of CPs *via* a hydrothermal route at a temperature of 250–300 °C for 2–5 days. Then, Goto^10^ used (Na, K)Al_2_Si_7_O_18_ as a raw material to obtain CP at 200 °C for 25 days in a weak alkaline solution at pH 7.9, but, a large number of associated phases such as mordenite were also formed. Although Satokawa and Itabashi^11^ synthesized Na-and K-CPs by crystallization for 6 days at 150 °C using the reactant system of 1.65 K_2_O : 1.65 Na_2_O : Al_2_O_3_ : 11 SiO_2_ : 275 H_2_O, high reaction temperature, and long crystallization time are not conducive for industrial production. Chi and Sand^12^ firstly used the “seed” method using natural CP as seeds (an additive amount of 1–10 wt%) to synthesis Na (K)-CPs with a relatively short crystallization time of 27–300 h at 120–195 °C. Subsequently, Zhao *et al.*^13^ studied the effect of various parameters such as alkali metals, silica–aluminum ratio, and alkalinity on the structure and texture properties of synthesized CPs. The results showed that Li-, Na-, K-, Rb-CPs can be successfully synthesized *via* a seed method, in which, the presence of potassium ions is beneficial to reduce the crystallization time. Williams *et al.*^14^ and Yuan *et al.*^15^ further elucidated the reproducible work of Chi and Sand's method,^12^ and found that it was still difficult to obtain highly pure CPs under laboratory conditions. Although the seed method actually expanded the range of starting compositions for the successful synthesis of CPs, the higher crystallization temperature, longer crystallization time, and lower crystallinity still could not satisfy the scale-up industries. Recently, Ouyang *et al.*^16,17^ proposed a structural promoter (SP) method to successfully synthesize highly pure CPs, which is a big difference from not only traditional structural directed agent but also the above-mentioned seed method.

On the basis of previous results and our preliminary work, the nanoprecursors including sol-SP, solid-SP, and solution-SP were synthesized *via* a hydrothermal route. The objective of this work is to explore the crystallization process of sol-SP particles and the effect of their additive amounts in the hydrothermal system on the structures and properties of synthetic CPs. Meanwhile, activation energies of crystallization kinetics of the CPs synthesized using additives consisting of various SPs were calculated during induction and growth periods, and therefore the promotion mechanism was put forward. The influences of various pre-crystallization times on the microstructure of the aluminosilicate sol and its morphology were emphasized, in which, the structural evolution over pre-crystallization time was explored using X-ray diffraction (XRD) patterns, nuclear magnetic resonance (NMR) spectra, Fourier transform infrared (FT-IR) and ultraviolet-visible (UV)-Raman spectra, scanning electron microscopic (SEM) images, thermogravimetric-differential scanning calorimetry (TG-DSC) analysis, and inductively coupled plasma (ICP). Subsequently, effects of pre-crystallization time and additive amounts on the obtained nanoprecursors were investigated in the hydrothermal system, the values of apparent activation energy of the induction period (*E*_n_) and growth procedure (*E*_g_) were calculated. Finally, the various synthetic CPs were used as adsorbents, their adsorption kinetics of CO_2_/CH_4_ and separation performances were preliminarily explored. These results showed that three kinds of SPs (sol-SP, solid-SP, and solution-SP) presented different mechanisms to promote the synthesis of CPs. The adsorption capacity and selectivity of CH_4_ and CO_2_ were evaluated, showing that the synthesized CPs had high adsorption capacity and CO_2_/CH_4_ separation ability. Different SPs did not affect the adsorption capacity of CPs, which initially indicated that it could be used as an efficient CO_2_/CH_4_ separation agent.

## 2 Experimental

### 2.1 Materials

As a silica source, an aqueous colloidal silica sol (Ludox JN-30, 30 wt% SiO_2_) with an average particle size of 10–20 nm and density of 1.2 g cm^−3^ supplied by Qingdao Ocean Chemical Plant was used. As an alumina source, Al(OH)_3_ (99.5 wt%) provided by Tianjin Fuchen chemical reagents factor was used. KOH (82.0 wt%) and NaOH (96.0 wt%) were purchased from Beijing Chemical Works. All the chemicals were of analytical reagent grade. The resistivity of the deionized water was 18.25 MΩ cm at 25 °C.

### 2.2 SPs preparation

According to our previous report,^16^ NaOH, KOH, Al(OH)_3_, and deionized water were placed in a 50 mL Teflon-lined beaker and stirred at 150 °C for 3 h until a transparent meta-aluminate solution (Na/K = 1) was obtained. Deionized water and silica sol were then added to the above prepared alkali metal–aluminate solution (Si/Al = 5.85, (Na + K)/Si = 0.48, H_2_O/SiO_2_ = 32.91). After stirring at room temperature for 2 h, the gel was transferred to a stainless-steel autoclave with polytetrafluoroethylene liners and kept at 150 °C for 6–102 h in the oven. Finally, the autoclave was taken out and cooled. The sol-SP was obtained and then partially centrifuged, in which, supernatant and precipitate were named as solution-SP and solid-SP, respectively.

### 2.3 Synthesis of CPs

NaOH, KOH, Al(OH)_3_, and deionized water were mixed in a 50 mL Teflon-lined beaker and stirred at 150 °C until it became a clear aluminate solution. Then, the remaining deionized water, silica sol, and sol-SP (or solution-SP, or solid-SP) with a designated amount were added to the above aluminate solution slowly, which was continuously stirred for 2 h at room temperature. After that, the prepared mixture was put into a Teflon-lined stainless-steel autoclave and further crystallized at 140–180 °C for 6–144 h. Finally, the autoclave was taken out and cooled, then by filtration and washing, the synthesized CPs were obtained.

It should be noted that the mass fraction of the added sol-SP (or solution-SP, or solid-SP) was approximately 1–18% of the synthesized mixture mass (excluding the mass of the sol-SP (or solution-SP, or solid-SP) required to be added). While the starting molar ratios of Na_2_O : K_2_O : SiO_2_ : Al_2_O_3_ : H_2_O were equal to 1.39 : 1.39 : 11.70 : 1: 385 in synthesized mixtures. The mass of each component of each sample in the starting synthesis system was collected in Table S1 of the ESI† section.

### 2.4 CO_2_/CH_4_ adsorption

First, 0.15 g of the above CPs were heated at 120 °C for 6 h in a vacuum to remove water and gases. Subsequently, CH_4_ or CO_2_ adsorption isotherms on each sample were measured at 0 or 25 °C with the retention pressure equal to the saturated vapor pressure of CO_2_ or CH_4_.

### 2.5 Characterizations

Crystal phases of the synthesized samples were determined using XD-6 (Beijing Purkinje General Instrument Co. Ltd) X-ray diffractometer with Cu Kα as the radiation source at 4°·min^−1^ and tested at 2*θ* range of 5–50°. The SEM instrument (JEOLJEM-220) operating at 15.0 kV was used to observe the morphology and structure of the synthesized samples. A 10 mg of sample was used during TG-DSC analysis on PerkinElmer Pyris I in the temperature range of 25–900 °C in the air atmosphere. The heating rate was 10 °C min^−1^ and the flow rate was 20 mL min^−1^. The FT-IR spectrum of the sample in the wavenumber range of 400–2000 cm^−1^ was recorded using an IR Prestige-21 FT-IR spectrophotometer. The elemental composition (Na^+^, K^+^, Si^4+^, and Al^3+^) and percentage of samples were determined using an ICP analyzer (Optima DV 2000), before analysis, the samples were dissolved in hydrofluoric acid (2.5%). The UV-Raman spectra of samples were measured in the wavenumber range of 200–1200 cm^−1^ using a Raman spectrometer (Lab Ram HR Evolution) with a laser source wavelength of 325 nm. The ^29^Si-NMR analysis was performed on an Agilent 600M solid-state nuclear magnetic resonance spectrometer with a resonance frequency of 99 MHz and MAS of 12 kHz, using TSP as the internal standard. Adsorption of CH_4_ or CO_2_ and adsorption–desorption isotherms of N_2_ was determined using JWGB jw-bk300 provided by Beijing Sci. & Tech. Co. Ltd. All samples were degassed for 6 h at a high vacuum of 120 °C, followed by measuring N_2_ adsorption–desorption isotherms at −196 °C. The Horvath–Kawazoe (HK) model was used to calculate micropore size distribution based on the desorption branch of isotherm.^18^ The micropore volumes of the synthesized CPs were calculated on the basis of the HK model using desorption data (relative pressure (*P*/*P*_0_) ≤ 0.20) of the N_2_ adsorption–desorption isotherms. While their inter-particle mesoporous volumes were calculated on the basis of the Barrett–Joyner–Halenda model *via* desorption data (0.20 ≤ *P*/*P*_0_ ≤ 0.99) of the N_2_ adsorption–desorption isotherms. Therefore, the total pore volumes of the synthesized CPs were equal to the sum of their micropore volume and the inter-particle mesopore volume.

## 3 Results and discussion

### 3.1 Structural characterization

**Fig. 1:**
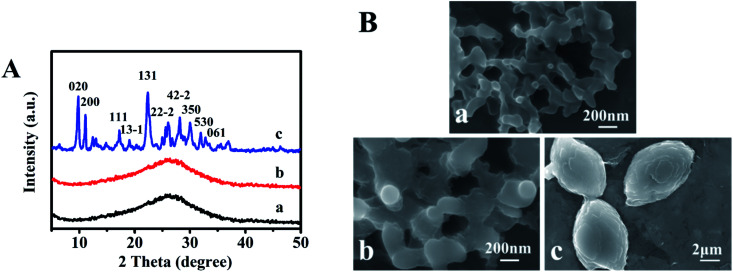
XRD patterns (A) and SEM images (B) of the obtained sol-SP with pre-crystallization time periods. (a) 6, (b) 60, and (c) 102 h.

Fig. 1A presents XRD patterns of sol-SP with different pre-crystallization times of 6, 60, and 102 h, respectively. As can be seen in Fig. 1A-a and -b, the samples with the pre-crystallization time of 6 and 60 h revealed diffuse peaks at 2*θ* of 10–40°, indicating the existence of amorphous phases. However, the characteristic peaks of CP, such as 9.9° (020), 11.2° (200), and 22.3° (131), appeared in the sol-SP that was pre-crystallized for 102 h (Fig. 1A-c), which can be used as an efficient “seeds” preliminarily.^16^ Correspondingly, Fig. 1B-a and -b show SEM micrographs, which revealed that the amorphous particles of sol-SP increased in size from 100 to 300 nm with the extension of pre-crystallization time of 6–60 h. While, the obvious CP particles with sheet stacked structures were obtained from sol-SP after crystallization for 102 h (Fig. 1B–c).

**Fig. 2:**
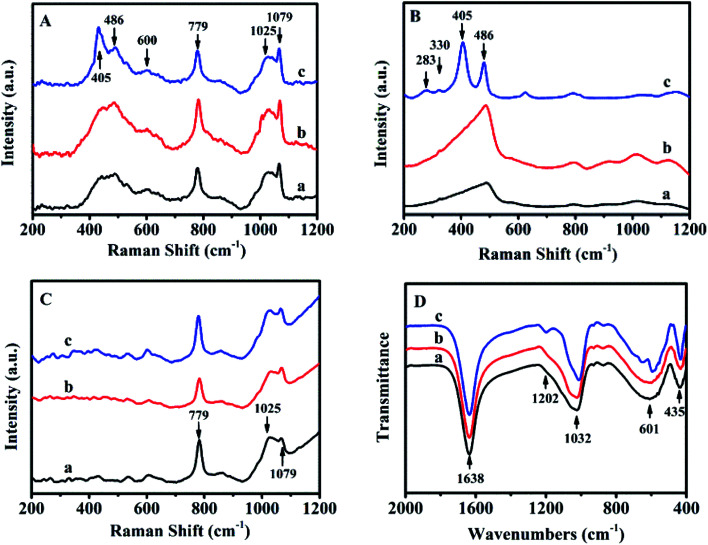
UV-Raman spectra of sol-SP (A), solid-SP (B), solution-SP (C) and FT-IR spectra of sol-SP (D) with pre-crystallization time periods. (a) 6, (b) 60, and (c) 102 h.

The sol-SP was further investigated by UV-Raman spectroscopy as shown from the data in Fig. 2A. As can be seen, Raman bands at 486 and 779 cm^−1^ could be assigned to monomeric silicate, the band at 600 and 1025 cm^−1^ was attributed to the oligomeric silicate species and another band at 1079 cm^−1^ belonged to double 4-membered rings.^19^ Therefore, the spectra of sol-SP with the pre-crystallization time interval of around 6 h (Fig. 2A-a) and 60 h (Fig. 2A-b) had the almost same composition, indicating the existence of main monomer and oligomeric silicate species. It can be speculated that these components of the initial crystallization may be composed of primary and secondary structural units of aluminosilicate. Correspondingly, FT-IR spectra also proved these observations (as shown in Fig. 2D). The peak at 1638 cm^−1^ was assigned to the deformation and vibration of H_2_O molecule,^20^ the bands at 1032 and 1202 cm^−1^ were attributed to the tetrahedral interior T–O–T (T = Si and Al) asymmetric stretching vibrations. While the others at 435 and 601 cm^−1^ were ascribed to the tetrahedron internal bending vibration of T–O and the external tetrahedron double loop.^21^

Fig. 2B shows the UV-Raman spectrum of solid-SP, which revealed that the strongest band at 486 cm^−1^ was due to the banding mode of the characteristic monomeric silicate,^19^ the bands at 283 and 330 cm^−1^ were corresponding to the banding mode of 8-membered rings (8R) and 6-membered rings (6R).^22^ While, the band at 405 cm^−1^ was assigned to the vibration of Si–O–Al in the framework of CP.^23^ However, the bands in the Raman spectra of solution-SP (Fig. 2C) were different from those of solid-SP (Fig. 2C). The strongest band at 779 cm^−1^ was attributed to the characteristic monomeric silicate, the band at 1025 cm^−1^ was assigned to the banding mode of the oligomeric silicate species, while the band at 1079 cm^−1^ belonged to the double 4-membered rings.^19^

These demonstrations suggest that the HEU structure may be formed in solid-SP, while the ring structure appeared in solution-SP, which could also be confirmed *via* FT-IR spectra. For example, in Fig. S1-A of the ESI section,† FT-IR spectra of the solid-SP mainly revealed bands at 1060, 1205 cm^−1^ (tetrahedral interior T–O–T (T = Si and Al) asymmetric stretching vibration), 449 cm^−1^ (tetrahedron internal bending vibration of T–O), and 606 cm^−1^ (external tetrahedron double loop).^21^ While, Fig. S1-B† indicated that FT-IR spectra of the solution-SP showed the bands at 1638 (deformation and vibration of H_2_O molecule),^20^ 1032 (tetrahedral interior T–O–T (T = Si and Al) asymmetric stretching vibration), and 601 cm^−1^ (external tetrahedron double loop).^21^

**Fig. 3:**
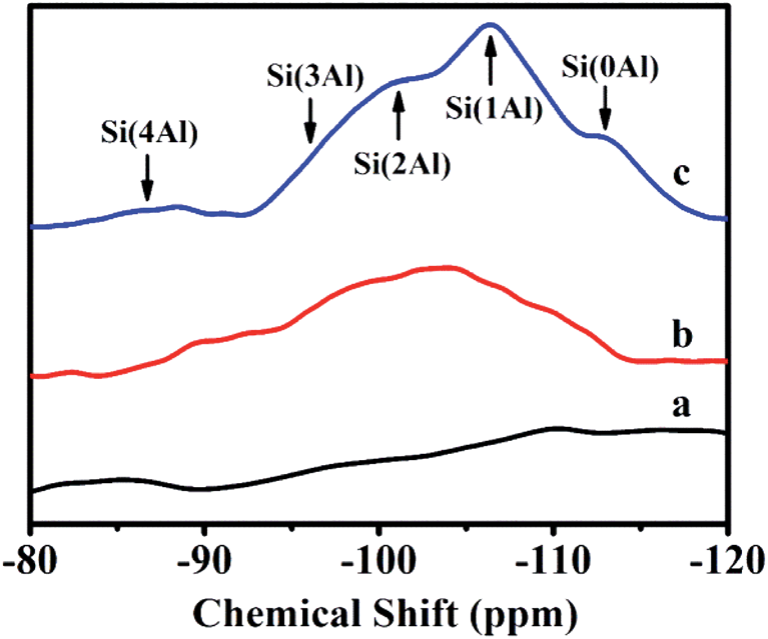
^29^Si-NMR spectra of solid-SP with pre-crystallization time periods. (a) 6, (b) 60, and (c) 102 h.

Fig. 3 illustrates ^29^Si-NMR profiles of solid-SP obtained at the pre-crystallization time of 6, 60, and 102 h, respectively. As can be seen, the broad resonances centered at −87, −95, −101, −106 and −114 ppm were characteristic of Si(4Al), Si(3Al), Si(2Al), Si(1Al) and Si(0Al) silicon environments. In the early stages of the crystallization of 6 h (Fig. 3a), the solid-SP structures were mainly Si(0Al) species, which may be due to the rapid combination of aluminate and silicate. These particles are highly chemically active, and thereafter are beneficial to promote the nucleation of aluminosilicate. With the extension of the pre-crystallization time up to 60 h (Fig. 3b), Si(0Al) is gradually transformed into Si(3Al) and Si(2Al). Subsequently, more silicates were embedded in the aluminosilicate networks and emerge the appearances of Si(1Al) mostly.^24,25^

In addition, the calculated Si/Al ratios (as shown in eqn (1) (ref. 26)) of solid-SPs increased with the prolongation of pre-crystallization time, showing 2.44 (6 h), 3.23 (60 h) and 4.84 (102 h), respectively, which was almost consistent with the reported literature.^27^1
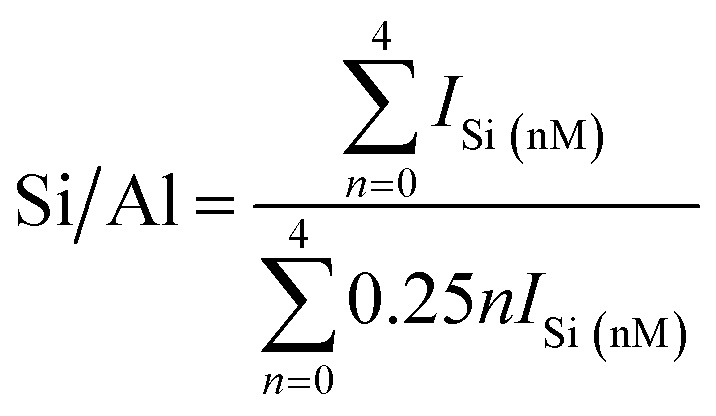
where *M* represents Al, *I*_Si (nM)_ represents the areas of each resonance and *n* is Si(*n*Al). Based on the above results, it is inferred that the crystallization behaviors of sol-SP are mainly involved in the following two processes: (1) amorphous and metastable precursors emerge during the early pre-crystallization stage, which is mainly composed of monomers and oligomeric silicate species, thereafter, the nucleus is generated by subsequent recombination or restructuring among the active Si(*x*Al)s [*x* = 0–4] species. (2) The amorphous aluminosilicates grow around the crystal nucleus so as to form crystal aggregates with a larger size.

**Fig. 4:**
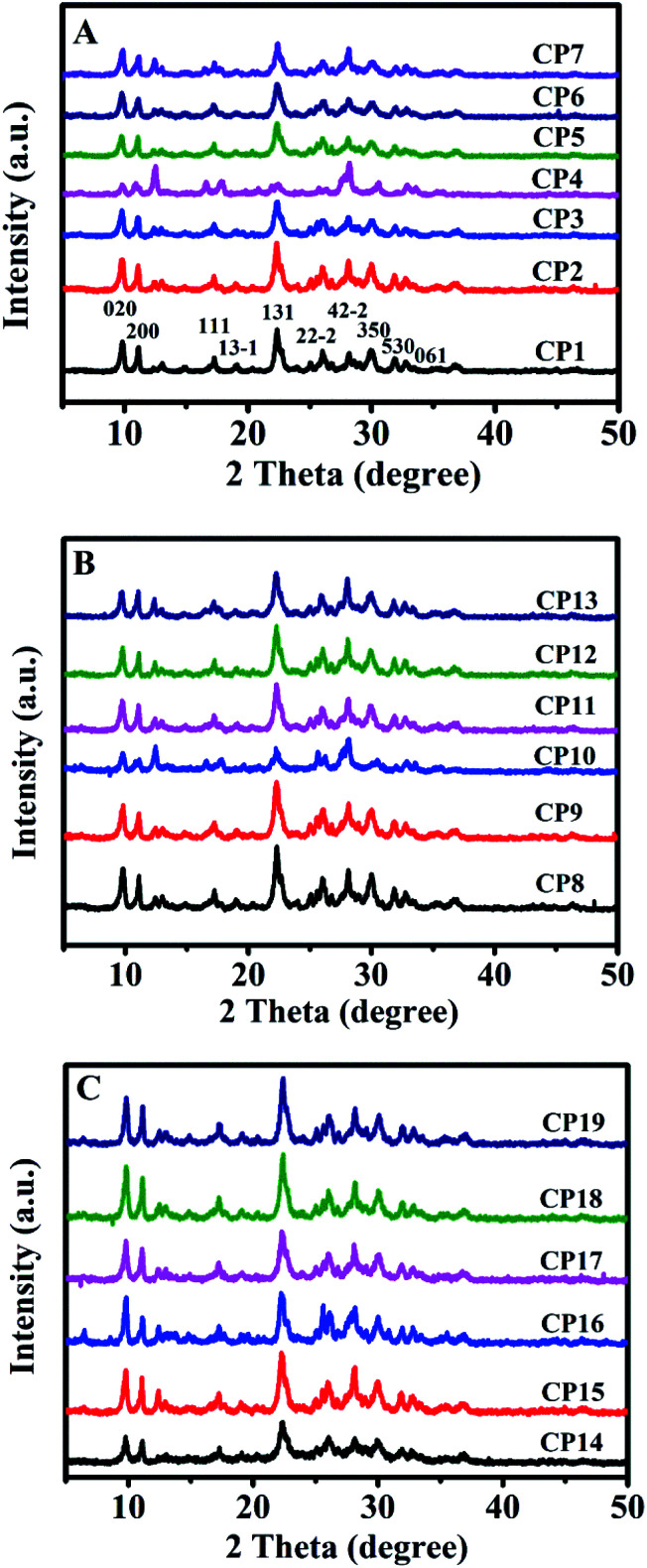
XRD patterns of synthetic CPs with additive sol-SP (A), solid-SP (B), and solution-SP (C).

Table 1 summarizes the various synthetic parameters of CPs and corresponding their phase compositions. Fig. 4A shows the XRD patterns of CPs synthesized with and without sol-SP. As can be seen in Fig. 4A, -CP1, -CP2, and -CP3, typical diffractive peaks of CP, such as (020), (200), (111), (13−1), (131), (22−2), (42−2), (350), (530) and (061),^28^ appeared in the synthesized samples without additive sol-SP or with the addition of pre-crystallization sol-SP for 6 and 60 h. However, as the pre-crystallization time of the sol-SP was extended to 102 h, a large number of coexisting phases, such as phillipsite and mordenite, appeared in the final products. In addition, Fig. 4A, CP3, -CP5, -CP6, and -CP7 indicated that the coexisting phases of phillipsite were more obvious with the increased additive amount of sol-SP.

**Table 1:** Summaries of various synthetic parameters of CPs and corresponding their phase compositions

Sample	Pre-crystallization time (h)			The additive amount of various SP^a^ (wt%)	Crystallization time (h)	Product phase	*S*_BET_ b	PV^c^	MPS^d^
	sol-SP	Solid-SP	Solution-SP						
CP1	—			0	144	cp	50.1	0.13	0.81
CP2	6			3	96	cp	—	—	—
CP3	60			3	84	cp	54.4	0.12	0.82
CP4	102			3	48	cp + phillipsite	23.8	0.06	0.92
CP5	60			6	78	cp + phillipsite	—	—	—
CP6	60			9	78	cp + phillipsite	—	—	—
CP7	60			18	66	cp + phillipsite	39.4	0.10	0.92
CP8		6		3	96	cp	—	—	—
CP9		60		3	84	cp	42.5	0.15	0.88
CP10		102		3	48	cp + phillipsite	23.9	0.07	0.95
CP11		60		6	78	cp + phillipsite	—	—	—
CP12		60		9	78	cp + phillipsite	—	—	—
CP13		60		18	66	cp + phillipsite	38.5	0.10	0.83
CP14			6	3	108	cp	—	—	—
CP15			60	3	108	cp	44.3	0.13	0.92
CP16			102	3	108	cp	41.3	0.12	0.96
CP17			60	6	108	cp	—	—	—
CP18			60	9	108	cp	—	—	—
CP19			60	18	108	cp	39.2	0.12	0.93

a The mass and composition of the synthetic SPs were the same as described in section 2.2.

b BET surface area (m^2^ g^−1^).

c Total pore volume (cm^3^ g^−1^).

d Mean micropore size (nm).

Similarly, as can be seen in Fig. 4B, XRD patterns suggested that the results obtained using the additive solid-SP were basically consistent with the same phenomena as that of the additive sol-SP (Fig. 4A). When the pre-crystallization time or the additive amount was increased, the occurrences of the impurity and other phases happened in the final products. However, different observations appeared for additive solution-SP, as shown in Fig. 4C, CP14, -CP15, and -CP16, the characteristic peaks of the synthesized samples gradually increased in intensity and no impurity phases appeared with the increase of the pre-crystallization time. Similarly, Fig. 4C, CP17, -CP18, and -CP19 presented that the synthesized samples revealed pure HEU structures without any impurity although the additive amount of solution-SP was increased when the pre-crystallization time of the solution-SP was the same.

**Fig. 5:**
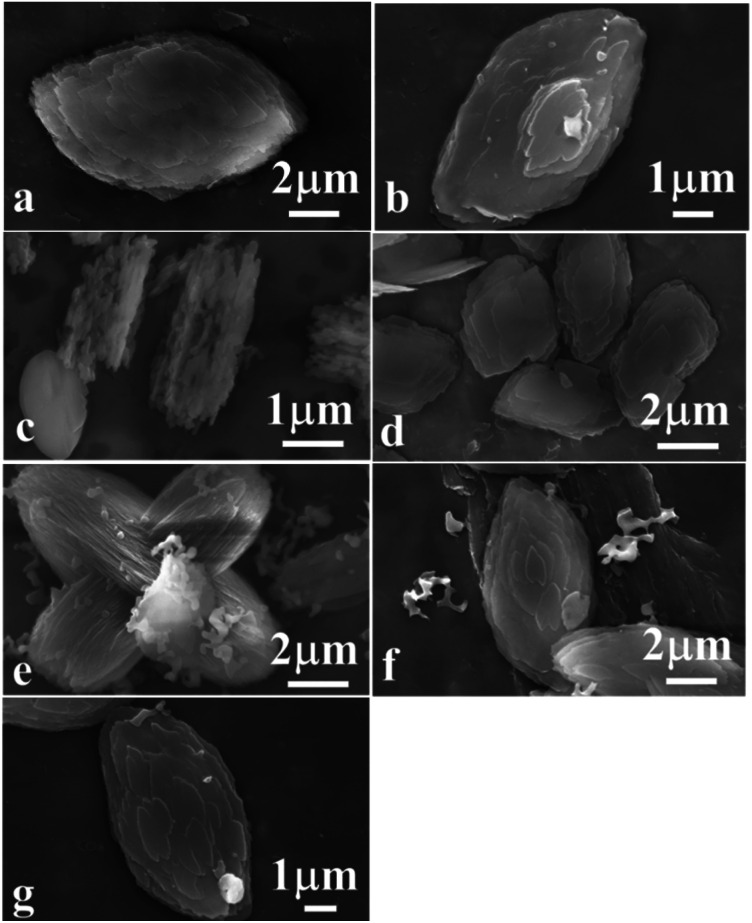
SEM images of synthetic CPs with various additive amounts of sol-SP, solid-SP and solution-SP. (a) CP1, (b) CP3, (c) CP4, (d) CP7, (e) CP15, (f) CP16, and (g) CP19.

Fig. 5 shows the morphologies of the synthetic CPs. First of all, Fig. 5a revealed the granular particles synthesized without additive SP in the size of about 10 μm, being consistent with the reported literature.^11,12^ Comparably, a significant decrease occurred in the particle size of CPs obtained in the presence of sol-SP. In detail, their particle sizes were about 7 and 2 μm when an additive amount (3 wt%) of pre-crystallization were from 60 and 102 h of sol-SP (as shown in Fig. 5b and c), respectively, similar to those obtained by the seed method.^12,13^ Meanwhile, their particle sizes also showed the declining tendencies with the increased additive amount (3–18 wt%) of pre-crystallization 60 h of sol-SP (as shown in Fig. 5b and d).

The observations of the samples synthesized with solid-SP were the same as those with sol-SP, showing the declining particle size with the increase in pre-crystallization time (as shown in Fig. S2-a and -b†) or the additive amount (as shown in Fig. S2-a and -c†). However, no matter what was the prolonged pre-crystallization time (as shown in Fig. 5e and f) or the increased additive amount of the solution-SP (as shown in Fig. 5e and g), the synthetic CPs presented granule morphology of size of around 8–10 μm. These phenomena obviously indicate that CPs with smaller granule size can be synthesized using sol-SP or solid-SP as additives, while the particle size and morphology of CPs synthesized using solution-SP have no obvious change.

Fig. S3† provides the N_2_ adsorption–desorption isotherms of all related samples, and their corresponding textural parameters are collected in Table 1. As can be seen, all the isotherms presented the H3-type hysteretic loops, which can be attributed to the multi-layer adsorption and capillary condensation phenomena.^7^ Meanwhile, a sudden increase in the adsorption capacity can be observed at very low pressure, indicating the presence of microporous structures with a mean pore size of approximately 0.8–0.9 nm. In addition, specific surface areas of synthetic CPs were slightly decreased with prolonged pre-crystallization time (such as CP3, CP4, and CP9, CP10, as shown in Table 1) or the increased additive amount of the sol-SP and solid-SP (such as CP3, CP7, and CP9, CP13, as shown in Table 1). One of the possible reason may be due to the presence of impurities in the synthetic CPs or the blockage of microspores. Comparably, the relative specific surface area of CPs synthesized with solution-SP had fewer impurities and higher crystallinity, the specific surface areas did not decrease significantly (such as for CP15, CP16, and CP19, as shown in Table 1).

### 3.2 Thermal stability (TG-DSC)

TG-DSC profiles of synthetic CPs were also collected and the results are provided in Fig. S4 of the ESI section.† As can be seen in Fig. S4-A,† the TG profiles can be divided into two stages, exhibiting continuous weight-loss tendencies as a function of temperature (25–900 °C). The first period at 25–300 °C had a high weight loss rate (7–9 wt%) and was generally considered to be the removal of physisorbed water,^29^ while, the second one during the temperature range of 300–500 °C presented a low weight loss of less than 2% at a slow rate, which was attributed to the dehydroxylation of the CPs.^30^ These results of additive sol-SP were basically the same as that of additive solid-SP (Fig. S4-B†) and solution-SP (Fig. S4-C†). While there was a slight difference in the adsorption capacities of the physisorbed water. Hence, these observations suggest that the additive SP has no obvious effect on the thermal stability of the synthetic CPs.

Meanwhile, two endothermic peaks of synthetic CPs in the temperature range of 25–900 °C were observed, as shown in DSC curves in Fig. S4† (inset). The first one at 25–300 °C was associated with the desorption of surface-adsorbed physical water, corresponding to a weight loss of the first stage in TG profiles. The second one at 300–500 °C may be related to the desorption of the combined water. However, an exothermic peak was not observed at higher temperatures, indicating that the phenomena of crystal transformation or structural collapse did not occur.

### 3.3 Crystallization kinetics

Based on the reported literature,^31–33^ the crystallization progress of traditional zeolites generally consists of three distinct regions: induction, growth, and stable periods, respectively. The research on zeolite synthesis mainly focuses on the induction period and growth period. The induction period mainly includes the nucleation process of crystals, and the time that approximately 10% crystallinity passes through is defined as the induction time in literature.^16^ During growth periods, the rapid evolution of the crystal structure results in a sudden change in the slope of the crystallization curve. The growth time is defined as the difference between the time taken to achieve constant crystallinity and the induction time. After that, the stable period mainly includes the process of slowing down the growth of the crystal. Therefore, according to the relative value of the sum of ten diffraction peaks in the XRD patterns of the CPs: (020), (200), (111), (13−1), (131), (22−2), (42−2), (350), (530), (061), the crystallinity of CPs obtained after the crystallized time of 144 h at 150 °C without SP were normalized as 1.0, and then the relative crystallinity of other samples was obtained accordingly.

**Fig. 6:**
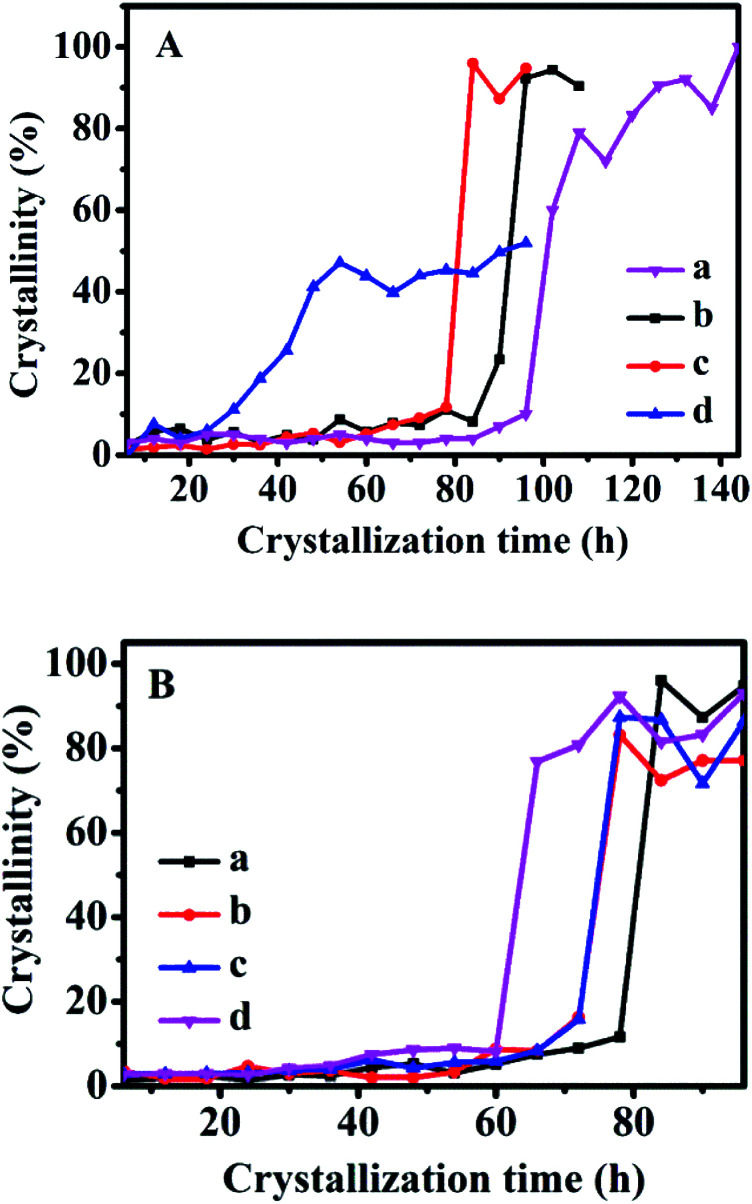
A: Crystallization kinetics of synthetic CPs without SP (a) or with additive 3 wt% of sol-SP at 150 °C and pre-crystallization time: (b) 6, (c) 60, and (d) 102 h. B: Crystallization kinetics of synthetic CPs with different amounts of sol-SP and pre-crystallization time of 60 h at 150 °C: (a) 3, (b) 6, (c) 9, and (d) 18 wt%.

Fig. 6 shows the crystallization kinetics of the synthetic CPs with the additive sol-SP. As can be seen, the induction time of the CPs synthesized without sol-SP was about 96 h (Fig. 6A-a), which was remarkably longer than that with the additive sol-SP. However, the induction time gradually decreased to 90, 78, and 24 h with the prolonged pre-crystallization time of 6 h (Fig. 6A-b), 60 h (Fig. 6A-c), and 102 h (Fig. 6A-d), respectively. Similarly, the crystal induction period declined to 78, 72, and 60 h with the increased additive amount of sol-SP of 3 (Fig. 6B-a), 6 (Fig. 6B-b), 9 (Fig. 6B-c), and 18 wt% (Fig. 6B-d).

Comparably, the addition of sol-SP did not significantly shorten the growth periods of the synthetic CPs, showing around 6–10 h. Obviously, the additive sol-SP is beneficial to the promotion of crystal nuclei but has little effect on the growth process. The possible reasons could be interpreted as follows: when the sol-SP is added to the synthesis system, it is rapidly combined with the amorphous aluminosilicate species in the mother liquor to generate the 8-member ring and 10-member ring structural units (as shown in Fig. 2), which consist of crystal nuclei. In this regard, the steps for controlling the reaction rate are directional polymerization of silicate and aluminate species, which is conducive to shortening the formation time of the crystal nucleus. Then, the recombination of crystal nuclei and aluminosilicate species led to the formation of nanocrystals, which subsequently aggregates the large crystals by polymerization, dissolution, and repolymerization.^34,35^ This explanation could be consistent with the crystallization process of conventional zeolites, such as FAU and L zeolite reported in the literature. For example, Kumar *et al.*^36^ studied the synthesis process of L zeolites and found that precursor particles were firstly formed in the crystallization process, and then gradually increased in size until reaching the maximum before the beginning of the growth period. However, the number of these particles continued to decrease after the beginning of the growth period, providing nutrients for the growth of L zeolites. Valtchev *et al.*^37^ believed that zeolite particles with small size and poor stability were easy to dissolve as nutrients, which promoted the formation of larger zeolite crystals in the growing period of FAU zeolite.

The crystallization kinetics of the synthetic CPs after adding solid-SP and shown in Fig. S5-A† is almost similar to that with sol-SP. The crystallization induction time of CPs was reduced to 90, 78, and 24 h with the increased re-crystallization time (6–102 h) of the additive solid-SP. Also, the induction times were shortened to 78, 72, and 60 h with the increase of additive solid-SP (3–18 wt%), respectively.

However, as can be seen in Fig. S5-B,† the crystallization kinetics of CPs synthesized by additive solution-SP presented that the induction time of the synthetic CPs was around 96 h, which showed a big difference from that with sol-SP and solid-SP, but very similar to that without additive SP (Fig. 6A-a). These investigations indicated that the solution-SP obtained either in crystallization time or in additive amount had little impact on the induction period. Meanwhile, the relative crystallinity of the synthetic CPs in the growth procedure varied slightly (around 90–97%) with additive solution-SP of different pre-crystallization times of 6, 60, and 102 h, higher than that 80% for CP synthesized without any additive (as shown in Fig. 6A-a). Similar phenomena on various additive amounts of solution-SP were also observed in Fig. S5-C.† Obviously, these results demonstrate that the additive solution-SP may be useful to promote the repaid growth of CPs after nucleation, but has no significant effect on decreasing the induction period.

**Fig. 7:**
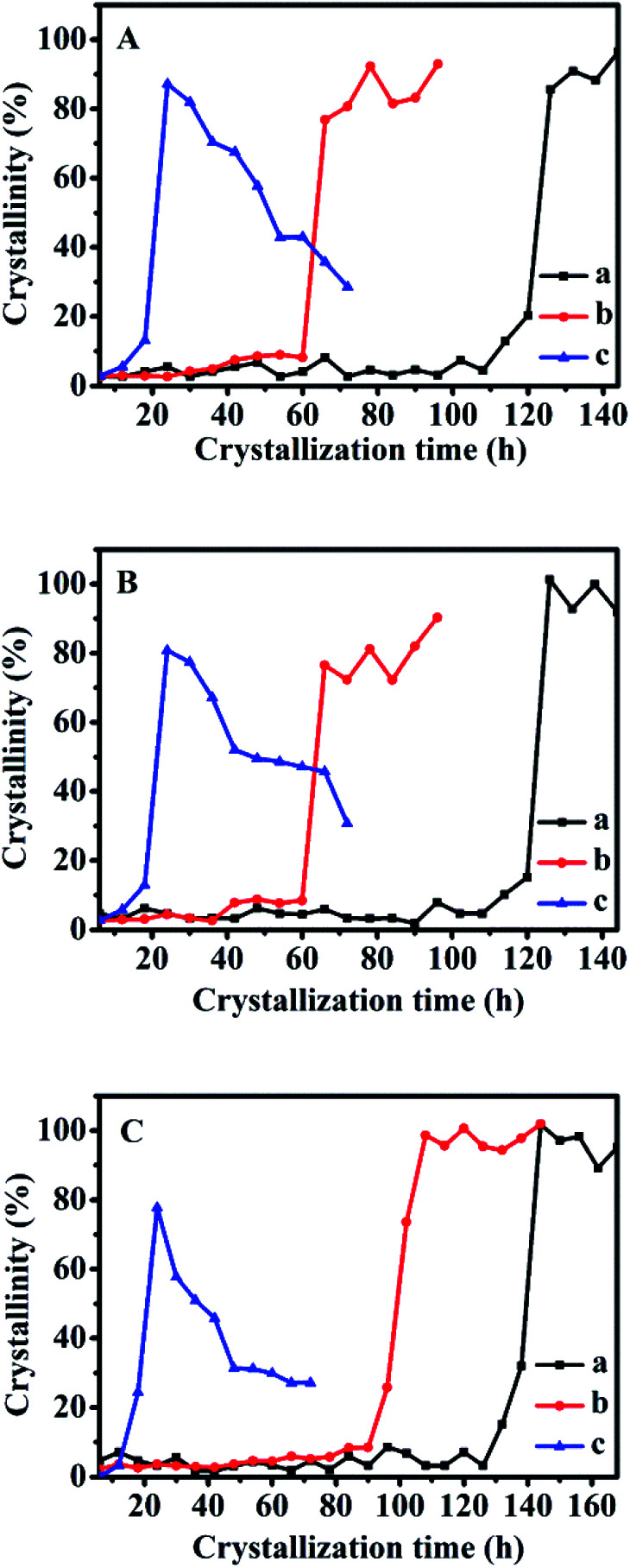
Crystallization curves for CPs synthesized with 18 wt% of sol-SP (A), solid-SP (B), and solution-SP (C) pre-crystallized for 60 h at different temperatures (a) 140 °C, (b) 150 °C, and (c) 180 °C.

The kinetic parameters of the crystallization process were further explored in detail as shown from the data in Fig. 7, representing the crystallization performances of CPs synthesized with additive 18 wt% of sol-SP, solid-SP, and solution-SP (pre-crystallization time of 60 h) at 140, 150, and 180 °C, respectively. The activation energy (*E*_n_ and *E*_g_) of each stage in the synthesis of CPs was calculated based on the Arrhenius equation.

The apparent *E*_n_ value was calculated using nucleation rate (1/*t*_0_) and temperature based on eqn (2).^38^2
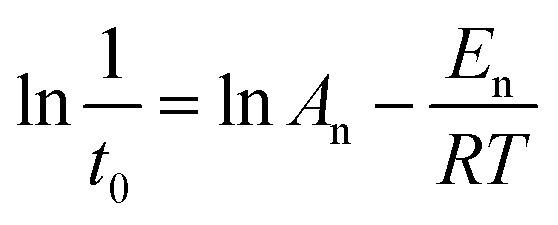


Similarly, eqn (3) was used to calculate the apparent *E*_g_ value,^39^ the rate constant (*k*) can be obtained from the slope at the steepest point of the crystallization curve.3
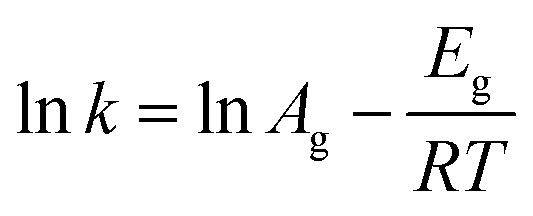


Therefore, the logarithmic graphs of the reciprocal of nucleation rate (or growth rate) and temperature are shown in Fig. S6 of the ESI section.†

Table 2 summarizes various parameters of *E*_n_, *t*_0_, and *k*_max_ values during crystallization of synthetic CPs. Based on the ICP data, chemical formulas of various synthetic CPs were provided, showing that the Si/Al molar ratio of related samples was about 5.79–6.44. As can be seen, the *E*_n_ value obtained by additive sol-SP was basically similar with the additive solid-SP that was around 65.5–67.0 kJ mol^−1^, but a little smaller than that (73.3 kJ mol^−1^) by the additive solution-SP, which was almost consistent with the result (73.9 kJ mol^−1^) in literature without any SP additive.^16^ Accordingly, *E*_g_ values obtained by additive sol-SP or solid-SP was almost the same, showing around 20.1–21.9 kJ mol^−1^, but larger than that (18.2 kJ mol^−1^) by additive solution-SP.

**Table 2:** Summaries of the chemical formula, *E*_n_, *t*_0_, and *k*_max_ values during crystallization of CPs

Sample	Chemical formula^a^	*T* (°C)	Induction period			Growth period		
			*t*_0_ (h)	ln *A*_n_	*E*_n_ (kJ mol^−1^)	*k*_max_	ln *A*_g_	*E*_g_ (kJ mol^−1^)
CP7	Na_1.15_K_5.55_Si_30.40_Al_5.23_O_72_	140	108	14.4	65.5	7.1	8.40	21.9
		150	60			9.0		
		180	18			13.0		
CP13	Na_1.32_K_4.56_Si_30.94_Al_4.79_O_72_	140	110	14.9	67.0	8.4	8.03	20.1
		150	60			10.2		
		180	18			14.3		
CP19	Na_1.16_K_4.19_Si_31.00_Al_4.88_O_72_	140	130	16.5	73.3	7.3	7.31	18.2
		150	85			8.1		
		180	18			11.6		

a CP1: Na_1.24_K_4.70_Si_30.85_Al_4.89_O_72_.

Therefore, the *E*_n_ value obtained by additive sol-SP was lower than that by additive solution-SP, suggesting that additive sol-SP can reduce the activation energy during the induction period and shorten the crystallization process. While, the results revealed that the *E*_n_ values are much higher than the *E*_g_ values, indicating that the induction period is the dominant kinetics during the crystallization process.

The results of the additive solution-SP show that it can promote the rapid growth of the synthetic CPs, the possible explanations are that the double four-member rings in the solutions should be connected to the crystal nucleus, resulting in the rapid growth of CPs. On the other hand, unlike the addition of sol-SP and solid-SP, the additive solution-SP had a less significant effect on the growth period, which could be caused by the relatively few active substances in the solution-SP.

### 3.4 Adsorption performances of CO_2_ and CH_4_

**Fig. 8:**
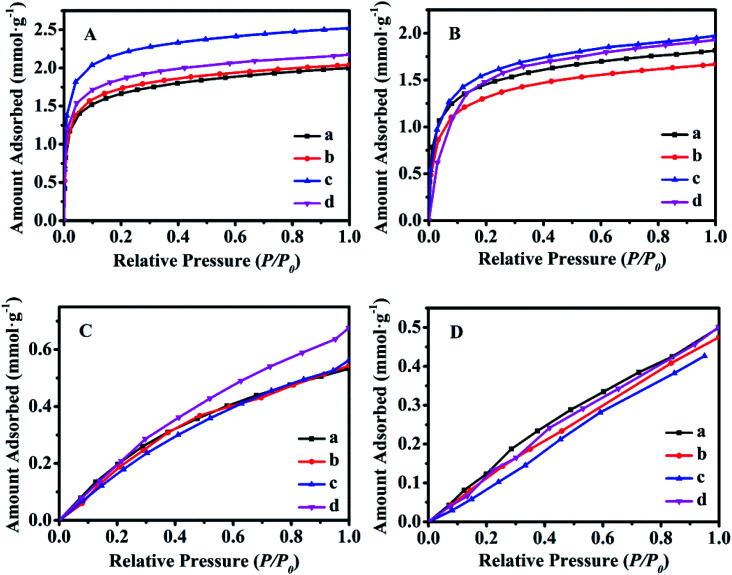
Equilibrium adsorbed isotherms of various synthetic CPs using CO_2_ as adsorbate at 0 °C (A) and 25 °C (B), respectively; CH_4_ as adsorbate at 0 °C (C) and 25 °C (D), respectively. (a) CP1, (b) CP7, (c) CP13, and (d) CP19.

Fig. 8 shows the equilibrium adsorption isotherms of CO_2_ and CH_4_ for various synthetic CPs at 0 and 25 °C. As can be seen, all samples presented the type I isotherms according to Brunauer's classification.^40^ The CO_2_ maximum adsorption capacities of CP1, CP7, CP13 and CP19 were up to 1.99, 2.04, 2.52, 2.17 mmol g^−1^ at 0 °C (Fig. 8A) and 1.81, 1.67, 1.97, 1.93 mmol g^−1^ at 25 °C (Fig. 8B), respectively. While, all of the related samples exhibited similar behavior for adsorbed CH_4_, showing the maximum capacities of around 0.53–0.67 mmol g^−1^ at 0 °C (Fig. 8C) and 0.47–0.50 mmol g^−1^ at 25 °C (Fig. 8D). However, their CO_2_ and CH_4_ uptake at 0 °C were much higher than that at 25 °C. Kennedy *et al.* also reported a similar result,^41^ the possible explanation is that the kinetic diameter (0.333 nm) of the CO_2_ molecule is smaller than that of CH_4_ (0.380 nm), which seems to easily diffuse into the micropore of CPs. Although the essence mechanism is still unclear, the charge distribution of exchangeable cations (Na^+^ and K^+^) in HEU skeletons also has a great influence on the adsorption performance.^7^

The Freundlich–Langmuir equation (as shown in eqn (4)) was used to calculate the molar adsorption capacity.^42^ Their adsorption heat of CO_2_ and CH_4_ could be also calculated on the basis of the Clausius–Clapeyron equation (as shown in eqn (5)).4
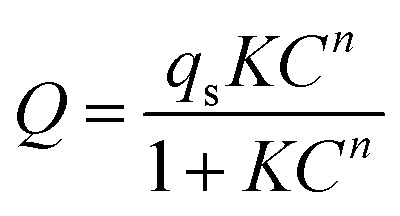
where *Q* is the adsorption capacity (mmol g^−1^) in moles; *q*_s_ is the molar adsorption capacity of the system (mmol g^−1^). *K* and *n* are constants, and *C* is relative pressure.5
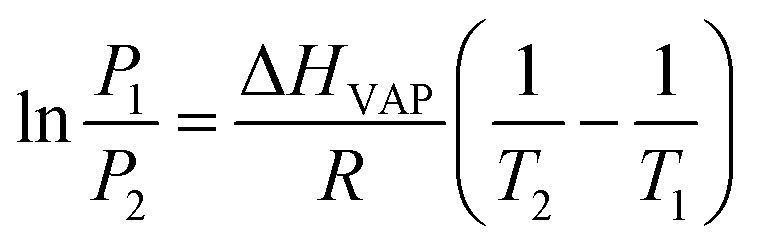
where *P*_1_ and *P*_2_ are the relative pressures under the temperatures of *T*_1_ and *T*_2_, Δ*H*_VAP_ is isochoric adsorption heat.

Various parameters were collected and are reported in Table S2.†

Accordingly, the selectivity and adsorption heat of CO_2_/CH_4_ is shown in Fig. S7.† As can be seen in Fig. S7-A and -B,† the adsorption calorific values of CO_2_ and CH_4_ adsorption were all greater than 0, indicating an endothermic process. The results were consistent with those described in the literature, for example, Salehi *et al.*^43^ and Davarpanah *et al.*^44^ calculated that adsorption heats of CP for CO_2_ and CH_4_ were 21.47 and 16.86 kJ mol^−1^, respectively, the reason for the low adsorption affinity is that CH_4_ and CO_2_ are non-polar molecules.

While Fig. S7-C and -D† exhibited similar performances of the CO_2_/CH_4_ selectivity at 0 and 25 °C. The CO_2_/CH_4_ selective factors for each sample (CP1, CP7, CP13, and CP19) were 3.42, 3.85, 4.82, 3.94 at 0 °C and 3.69, 3.51, 4.45, 3.90 at 25 °C, respectively. Compared with CO_2_/CH_4_ selective adsorption performance for reported materials (as shown in Table S3†),^43,45–48^ it can be speculated that the separation performance of CPs can be further improved *via* Li^+^, Ca^2+^, Ce^3+^ modifications. These results implied that the synthesized CPs should be a promising candidate for CO_2_/CH_4_ separation.

## 4 Conclusions

The different pre-crystallized sol-SP, solid-SP, and solution-SP were prepared, which were used as structure promoters to be added to the hydrothermal system for the successful synthesis of three types of CPs. Various characterizations demonstrated their differences in composition and structural properties. The Arrhenius equation was employed to calculate activation energy values (*E*_n_ and *E*_g_) of the induction phase and growth phase during the synthesis processes. The adsorption capacity and its separation ability of synthetic CPs for CO_2_ or CH_4_ were preliminarily explored. The results showed that the nucleation process is the main controlling step during the synthesis of CPs, whereas sol-SP and solid-SP can both promote the formation of crystal nuclei and shorten the crystallization time, showing the same promotion mechanism. Particularly, with an increased additive amount of sol-SP (solid-SP) or prolonging of their pre-crystallization time, the *E*_n_ value during the induction period decreased gradually, however, either too long pre-crystallization time or too much addition easily led to appearances of the impurity phase (phillipsite) in synthetic CPs. Comparably, solution-SP accelerated the growth process in the synthesis of CPs, which was beneficial to improve the relative crystallinity of CPs but had no obvious effect on the induction period. The adsorption capacity and separation performance of the synthetic CPs for CO_2_/CH_4_ provided a theoretical basis for the further design, synthesis and modification of better CP adsorbents.

## Author contributions

Chengwei Zhai: investigation, writing-original draft preparation, Bingying Jia, Anadil Gul: data curation, Jihong Sun: supervision, conceptualization, methodology, Shiyang Bai: formal analysis, validation.

## Conflicts of interest

There are no conflicts to declare.

## Supplementary Material

## References

[cit1] Alver B. E., Sakizci M. (2015). Adsorption.

[cit2] Jayaraman A., Hernandez-Maldonado A. J., Yang R. T., Chinn D., Munson C. L., Mohr D. H. (2004). Chem. Eng. Sci..

[cit3] Kennedy D. A., Khanafer M., Tezel F. H. (2019). Microporous Mesoporous Mater..

[cit4] Frankiewicz T. C. and Donnelly R. G., in Industrial Gas Separations, ed. T. E. Whyte, C. M. Yon and E. H. Wagener, American Chemical Society, Washington D.C., 1983, vol. 223, ch. 11, pp. 213–233

[cit5] Chao C. C. , *US Pat.*, 4964889, 1990

[cit6] Aguilar-Armenta G., Hernandez-Ramirez G., Flores-Loyola E., Ugarte-Castaneda A., Silva-Gonzalez R., Tabares-Munoz C., Jimenez-Lopez A., Rodriguez-Castellon E. (2001). J. Phys. Chem. B.

[cit7] Kouvelos E., Kesore K., Steriotis T., Grigoropoulou H., Bouloubasi D., Theophilou N., Tzintzos S., Kanelopoulos N. (2007). Microporous Mesoporous Mater..

[cit8] Güvenir Ö., Kalıpçılar H., Çulfaz A. (2009). Cryst. Res. Technol..

[cit9] Ames Jr L. (1963). Am. Mineral..

[cit10] Goto Y. (1977). Am. Mineral..

[cit11] Satokawa S., Itabashi K. (1997). Microporous Mater..

[cit12] Chi C. H., Sand L. (1983). Nature.

[cit13] Zhao D., Szostak R., Kevan L. (1998). J. Mater. Chem..

[cit14] Williams C. D. (1997). Chem. Commun..

[cit15] Yuan J. S., Shi L., Han H. R., Ji Z. Y. (2007). Chin. J. Inorg. Chem..

[cit16] Ouyang T., Zhai C. W., Sun J. H., Panezai H., Bai S. Y. (2020). Microporous Mesoporous Mater..

[cit17] Sun J. H. , Ouyang T., Bai S. Y., Zhai C. W., Jiao J. and Li J., Chin. Appl. Pat., CN109592696A, 2019

[cit18] Musa M. A. A., Yin C. Y., Savory R. M. (2010). Mater. Chem. Phys..

[cit19] Zhao X., Liu R., Zhang H., Shang Y., Song Y., Liu C., Wang T., Gong Y., Li Z. (2017). J. Appl. Crystallogr..

[cit20] Corral-Capulin N. G., Vilchis-Nestor A. R., Gutiérrez-Segura E., Solache Ríos M. (2018). J. Fluorine Chem..

[cit21] Garcia-Basabe Y., Rodriguez-Iznaga I., Menorval L. C. D., Llewellyn P., Maurin G., Lewis D. W., Binions R., Autie M., Ruiz-Salvador A. R. (2010). Microporous Mesoporous Mater..

[cit22] Xiao Y., Sheng N., Chu Y., Wang Y., Wu Q., Liu X., Deng F., Meng X., Feng Z. (2017). Microporous Mesoporous Mater..

[cit23] Elghniji K., Elaloui E., Moussaoui Y. (2017). Chem. Pap..

[cit24] Rivera A., Farıas T., Ruiz-Salvador A., Ménorval L. C. D. (2003). Microporous Mesoporous Mater..

[cit25] Lippmaa E., Maegi M., Samoson A., Tarmak M., Engelhardt G. (1981). J. Am. Chem. Soc..

[cit26] Al-Yassir N., Akhtar M. N., Al-Khattaf S. (2012). J. Porous Mater..

[cit27] Tanaka H., Yamasaki N., Muratani M., Hino R. (2003). Mater. Res. Bull..

[cit28] Treacy M. M. J. and Higgins J. B., Collection of simulated XRD powder patterns for zeolites, Elsevier Science, Amsterdam, 2001

[cit29] Yrükoullar E., Yılmaz G., Dikmen S. (2010). J. Therm. Anal. Calorim..

[cit30] Alver B. E., Sakizci M., Yörükoğullari E. (2009). J. Therm. Anal. Calorim..

[cit31] Nikolakis V., Vlacho D. G., Tsapatsis M. (1998). Microporous Mesoporous Mater..

[cit32] Dong J. L., Xu Q. H., Ryong C. H. (1993). Chin. J. Inorg. Chem..

[cit33] Peng S. L., Ullah R., Bai S. Y., Sun J. H., Wu X. (2019). Acta Petrol. Sin..

[cit34] Oleksiak M. D., Soltis J. A., Conato M. T., Penn R. L., Rimer J. D. (2016). Chem. Mater..

[cit35] Mintova S., Olson N. H., Valtchev V. P., Bein T. (1999). Science.

[cit36] Kumar M., Li R., Rimer J. D. (2016). Chem. Mater..

[cit37] Valtchev V. P., Bozhilov K. N. (2004). J. Phys. Chem. B.

[cit38] Marzpour Shalmani F., Halladj R., Askari S. (2016). Ultrason. Sonochem..

[cit39] Uzcátegui D., González G. (2005). Catal. Today.

[cit40] Brunauer S., Deming L. S., Deming W. E., Teller E. (1940). J. Am. Chem. Soc..

[cit41] Kennedy D. A., Mujčin M., Abou-Zeid C., Tezel F. H. (2019). Microporous Mesoporous Mater..

[cit42] Yang X., Epiepang F. E., Li J. B., Wei Y. W., Liu Y. S., Yang R. T. (2019). Chem. Eng. J..

[cit43] Salehi R. N., Sharifnia S., Rahimpour F. (2018). J. Nat. Gas Sci. Eng..

[cit44] Davarpanah E., Armandi M., Hernández S., Fino D., Arletti R., Bensaid S., Piumetti M. (2020). J. Environ. Manage..

[cit45] Arefi Pour A., Sharifnia S., NeishaboriSalehi R., Ghodrati M. (2015). J. Nat. Gas Sci. Eng..

[cit46] Oddy S., Poupore J., Tezel F. H. (2013). Can. J. Chem. Eng..

[cit47] Kennedy D. A., Tezel F. H. (2018). Microporous Mesoporous Mater..

[cit48] Montes Luna A. D. J., Castruita de León G., García Rodríguez S. P., Fuentes López N. C., Pérez Camacho O., Perera Mercado Y. A. (2018). J. Nat. Gas Sci. Eng..

